# Stigmasterol alleviates airway inflammation in OVA-induced asthmatic mice via inhibiting the TGF-β1/Smad2 and IL-17A signaling pathways

**DOI:** 10.18632/aging.205716

**Published:** 2024-04-04

**Authors:** Sihong Huang, Rong Zhou, Yuyun Yuan, Yiyun Shen

**Affiliations:** 1 Department of Pediatrics, Shanghai Baoshan District Hospital of Integrated Traditional Chinese and Western Medicine, Shanghai University of Traditional Chinese Medicine, Shanghai 201999, China

**Keywords:** asthma, stigmasterol, Th-17, TGF-β1, Smad2, IL-10

## Abstract

Stigmasterol is a common dietary phytosterol with high nutritional value and physiological activity. In this study, we evaluated the effects of stigmasterol on inflammatory cytokines and the TGF-β1/Smad2 and IL-17A signaling pathway in an ovalbumin (OVA)-induced asthma mouse model. Stigmasterol treatment improved airway remodeling. In addition, it significantly attenuated the symptoms of asthma attacks, reduced the number of macrophages, lymphocytes, neutrophils, and eosinophils in BALF and inflammatory cytokines, including IL-1β, IL-5, IL-6, and IL-13. It further decreased the level of IL-17A in BALF, serum and spleen. Spleen single-cell suspension analysis via flow cytometry showed that IL-17A level was consistent with the results obtained in BALF, serum and spleen. Stigmasterol decreased the protein expression levels of TGF-β, p-Smad2 and IL-17A in the spleen, by increasing the protein expression level of IL-10. After 24 h of co-culture of TGF-β, IL-6 and stigmasterol, the level of IL-17 in CD4^+^ T cell supernatant was lower relative to levels in the group without stigmasterol. Meanwhile, stigmasterol treatment attenuated the expression level of TGF- β, p-Smad2 and IL-17A proteins in CD4^+^ T cells and enhanced the expression levels of IL-10 protein. These data suggested that stigmasterol inhibited the TGF-β1/Smad2 and IL-17A signaling pathway to achieve anti-asthmatic effects in the OVA-induced asthma mouse model. Collectively, the results of this study are that stigmasterol has achieved preliminary efficacy in the non-clinical laboratory, further studies are needed to consider the clinical application of stigmasterol.

## INTRODUCTION

Stigmasterol is a natural phytosterol found in many plant fats. It is used as an active ingredient in many medicinal plants, vegetables and nuts [1]. Structurally, it is similar to animal sterols [2]. It is widely applied in pharmaceutical, food and cosmetic industries due its proven pharmacological effects, including anti-inflammatory and anti-allergic, immunomodulatory effects [3]. For example, stigmasterol treatment reduced TNF-α, IL-6, IL-1β, iNOS and COX-2 in collagen-induced rheumatoid arthritis (RA) rats, and increased the expression of anti-inflammatory cytokines (IL-10) by down-regulating NF-kB p65 and p38 MAPK expression in the joints [4].

Asthma is a heterogeneous chronic airway inflammatory disease characterized by airway inflammation, reversible airway obstruction and airway hyperresponsiveness [5]. It affects 1–18% of the world's population globally [6]. Macrophages, mast cells, lymphocytes, neutrophils, eosinophils, airway epithelial cells, and their components play a role in the pathological processes of asthma [7]. Although the pathomechanisms of asthma are not clear, inflammatory response is considered to play an important role, and suppression of the inflammatory response is therefore an effective strategy to treat asthma [8, 9].

Once external allergens enter the body, they are taken up by macrophages and processed to form MHCII-antigen peptide complexes, which are specifically recognized by the initial CD4^+^ T (Th 0) cells, causing disruption of the balance in Th0 cell differentiation [10]. It is well known that Th1/Th2 immune dysregulation is a common feature of asthmatic airway inflammation [11, 12] and that cytokines IL-4, IL-5, IL-6 and IL-13 produced by Th2 cells play an important role in asthma pathogenesis [13]. IL-4 and IL-13 promote the completion of IgE antibody type switching by B lymphocytes; IL-5 increases eosinophilia; IL-13 is associated with abnormal proliferation of goblet cells, mucus hypersecretion and airway hyper reactivity (AHR) [14]. In this process, activation of macrophages increases the production of cytokines IL-6 and IL-1β. As typical inflammatory cytokines, IL-1 β and IL-6 have also been used as indicators to diagnose various inflammatory experiments [15]. The accumulation of eosinophils and induction of the conversion of immunoglobulin classes to IgE participates in eosinophilic asthma. They are also involved in the regulation of mucus hypersecretion and airway hyperresponsiveness in asthma, causing a significant increase in IL-17, accompanied by a decrease in IL-10 in neutrophilic asthma [16]. Apart from Th2 cytokines, attention has been paid to the role of Th17 cytokines in the development of severe asthma. There is growing evidence that IL-17A contributes to the development of asthma, particularly severe asthma characterized by intense neutrophil infiltration of the airways. Clinical studies have shown that IL-17A is significantly increased in bronchoalveolar lavage fluid (BALF) and serum of asthma patients compared to healthy people. Furthermore, there is a positive correlation between IL-17A levels and disease severity [17]. Currently, several scientific reports have found that the TGF-β/Smad signaling pathway also regulates the differentiation process of Th17 cells [18], suggesting that inhibiting IL-17A may be an effective anti-asthma avenue.

In this study, we explored therapeutic effects of stigmasterol on asthmatic mice. Additionally, the role of stigmasterol was also investigated *in vitro* in isolated CD4^+^ T cells. The present results reveal that stigmasterol is a potential treatment for asthma-related symptoms [19].

## MATERIALS AND METHODS

### Asthma model and treatment

In the study, C57/BL6 male mice (6 weeks, 20 ± 2 g) were obtained from SLAC Laboratory Animal Co. Ltd., (Shanghai, China) and housed under specific pathogen-free conditions. This study was carried out in strict accordance with the Guide for the Care and Use of Laboratory Animals (Eighth Edition, 2011, published by The National Academies Press, Washington, DC, USA). The protocol was reviewed and approved by the Shanghai University of Traditional Chinese Medicine Institutional Review Board (Permit Number: HKDL2020348), and all animal experiments were conducted in Shanghai Seventh People’s Hospital. All surgeries were performed under sodium pentobarbital anesthesia, and all efforts were made to minimize suffering.

**Figure 1:**
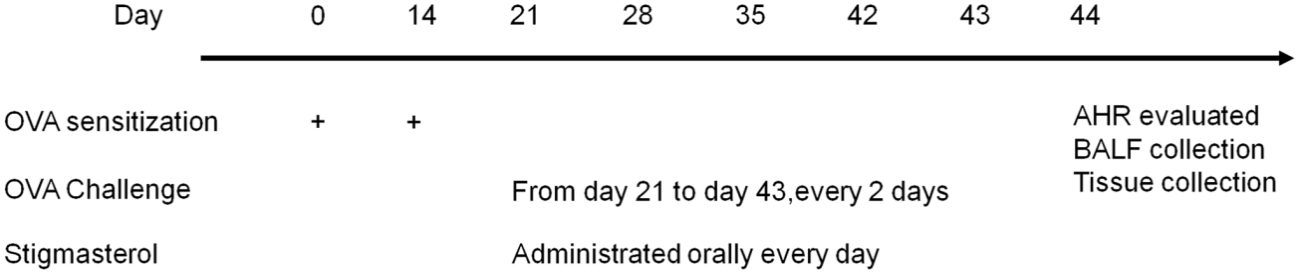
Flow chart of animal specific time node experiments.

The mice were randomly assigned to six groups, six mice in each group: (1) Control; (2) OVA (ovalbumin) [20]; (3) OVA + Stigmasterol (STG) (5 mg/kg); (4) OVA + STG (10 mg/kg); (5) OVA + STG (20 mg/kg); mice in the control group were challenged with aerosolized saline and exposed to room air. In the asthma model, the mice were sensitized intraperitoneally (i.p.) with 20 μg OVA (Sigma-Aldrich, St. Louis, MO, USA) complexed with 2 mg of alum (Shanghai No. 4 Reagent and H.V. Chemical Industries, Ltd., Shanghai, China) in a total volume of 0.1 ml saline on day 0 and day 14. The mice were challenged via aerosol nebulization with 5% OVA (w/v) for 30 min every 2 days from day 21 to day 43 (the control mice received saline). The mice in the OVA-challenged received stigmasterol. Stigmasterol (Yuanye Bio-Technology, Shanghai, China) was administrated orally. The study protocol is shown in Figure 1. All mice survived in a standard SPF environment, in which the temperature was 22 ± 2°C, the humidity was 40% to 70%, and the air inside the barrier was cleaned to class 10,000. All animal experiments were conducted in Shanghai Seventh People’s Hospital. All surgeries were performed under sodium pentobarbital anesthesia, and all efforts were made to minimize suffering.

### Preparation of BALF [21]

24 h after the last chronic IH exposure, mice were sacrificed by an overdose administration of pentobarbital (100 mg/kg intraperitoneal), and the lungs were isolated by blunt dissection. Three successive volumes of 1 ml phosphate-buffered saline were instilled via the endotracheal tube and aspirated gently, and bronchoalveolar lavage fluid (BALF) was pooled. Each BALF sample was centrifuged at 1000 g for 10 min at 4°C, and the supernatants were stored at −80°C until use. Cell pellets were diluted with 0.5 ml of phosphate-buffered saline. Total cell counts were determined using a hemocytometry by adding 100 μl of the cell suspension to 100 μl of 0.4% trypan blue. Differential cell counts were performed on cytocentrifuge preparations (Cytospin 2; Shandon Instruments, Runcorn, UK) stained with Wright-Giemsa, and 200 cells were counted under ×400 magnification by three different investigators in a blinded manner. Cells were identified based on standard morphology and classified as macrophages, lymphocytes, neutrophils, and eosinophils. The levels of inflammatory cytokines interleukin IL-6, IL-5, IL-13 and IL-1β in BALF were determined using an ELISA kit (Abcam, Cambridge, UK) according to standard protocols.

### Preparation of mouse spleen cell suspension

The mice were sacrificed through cervical dislocation, the spleen was removed aseptically, the spleen tissue was cut on the ultra-clean bench, lightly ground in a 200-mesh sieve, the sieve was rinsed with serum-free RPMI-1640 medium, and the splenocyte precipitate was collected by centrifugation at 1500 r/min for 5 min at 4°C. Add 2 ml of erythrocyte lysate, mix thoroughly, and reacted at room temperature for 5 min with intermittent gentle shaking. Add about 10 ml of RPMI-1640 to terminate the lysis, mix well, centrifuge and discard the supernatant, and the resulting cells are splenic single nucleated. Confirm that the percentage of live cells exceeds 95%, adjust the final concentration of cells to 1 × 10^6^/ml, and set aside.

### Isolation of splenic CD4^+^ T cells

After obtaining the spleen single-cell suspension, pre-chilled phosphate buffered saline (PBS) was added for cleaning, centrifuged again to collect the precipitation, pre-chilled PBS was added to form the suspension. Then, 1.0 × 10^8^ cells were transferred to a 15 ml Corning tube and centrifuged at 4°C 1000 rpm for 5 min. The cell precipitate was retained and suspended in 5 ml buffer (PBS + 3% fetal bovine serum), and then 100 μL of biotin–antibody cocktail was added immediately. The mixture was allowed to interact at 4°C for 1 h. Finally, add 300 μL buffer and 200 μL MicroBeads were mixed and allowed to interact at 4°C for 30 min. The mixture was centrifuged at 1000 rpm at 4°C for 5 min, the precipitate was collected and resuspended at 500 μL buffer. The suspension flowed into the LS column for collecting the filtrate. This process needed to be repeated several times for collecting the pure filtrate. The filtrate was resuspended to get CD4^+^ T cells.

### Statistical analysis

The data were expressed as mean ± SD (Standard Deviation). Each experiment was repeated at least 3 times. Statistical analysis was performed by using one-way ANOVA and R language. *P* < 0.05 was considered statistically significant.

### Data statement

The data that support the findings of this study are available from the corresponding author upon reasonable request.

## RESULTS

### Stigmasterol improved the inflammatory response in the airways of asthmatic mice

**Figure 2:**
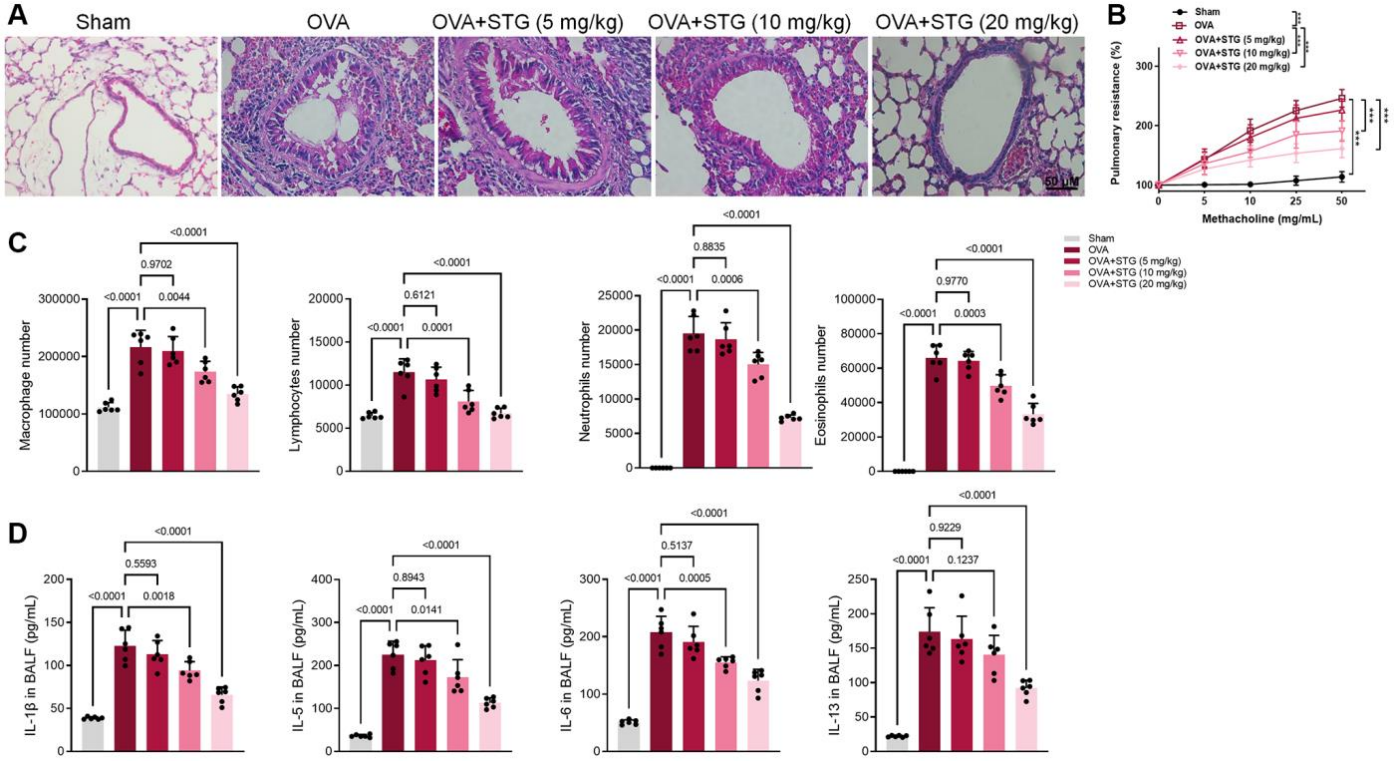
**Effect of stigmasterol on OVA-challenged mice airway inflammation and AHR.** (**A**) Histological H&E staining of airway remodeling, ×200. (**B**) STG reduced OVA-induced airway hyperresponsiveness. The effects of STG on AHR were detected by an invasive pulmonary facility for small animals (FlexiVent, SCIREQ, USA). Data are expressed as a percentage change from the baseline value. (**C**) The number of inflammatory cells such as macrophages, lymphocytes, neutrophils, and eosinophils in BALF as detected by flow cytometry. (**D**) Inflammatory factors in BALF including IL-1β, IL-5, IL-6, IL-13 as determined by ELISA. *p* < 0.05 indicated statistically significant differences.

Mice were intranasally challenged with or without OVA and then sacrificed 48 h after the last challenge. Inflammatory cells were recruited to the lungs of the mice were also investigated by histopathological studies. Compared with the control group, OVA-challenged mice showed thickening of airway smooth muscle and infiltration of a large number of inflammatory cells around the bronchus and blood vessels. However, that symptom was significantly reversed in the stigmasterol-treated mice, which was compared to the OVA-challenged mice (Figure 2A). Further analysis showed that AHR to methacholine was significantly increased in OVA-challenged mice compared to control mice (*p* < 0.01), and significantly reduced in 10 mg/kg or 20 mg/kg of stigmasterol-treated mice compared to OVA-challenged mice (*p* < 0.001) (Figure 2B). In OVA-challenged mice, the numbers of macrophages, lymphocytes, neutrophils, eosinophils and were increased in BALF. However, a decrease occurred after soy steroid treatment and there was a dose-dependent laziness, with significant effects at 10 mg/kg and 20 mg/kg soy steroid (Figure 2C). The supernatant of BALF was examined by ELISA, and it was found that IL-1β, IL-5, IL-6 and IL-13 in BALF of mice subjected to OVA showed a significant increase, and IL-1β, IL-5 and IL-6 were significantly down-regulated after 10 mg/kg and 20 mg/kg soy steroid treatment, and IL-13 was significantly down-regulated after 20 mg/kg stigmasterol treatment (Figure 2D).

### Stigmasterol reduced IL-17A levels and increased IL-10 levels in BALF, serum, and splenocytes of OVA-challenged mice

**Figure 3:**
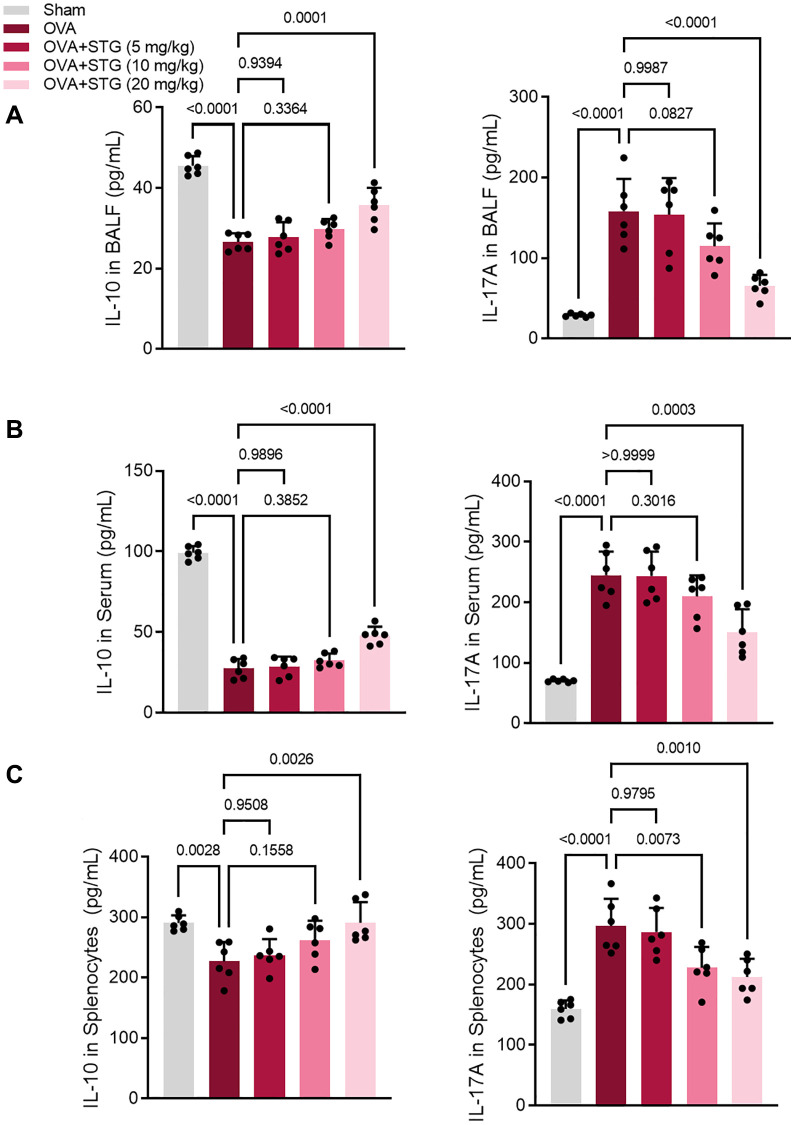
**Effect of maltol on IL-17A and IL-10 expression in BALF, serum and splenocytes of OVA-challenged mice.** (**A**) Expression levels of IL-17A and IL-10 in BALF. (**B**) Expression levels of IL-17A and IL-10 in serum. (**C**) Expression levels of IL-17A and IL-10 in splenocytes. *p* < 0.05 indicated statistically significant differences.

IL-17A levels were significantly higher in BALF, serum, and splenocytes samples from mice in the OVA challenge group compared with the control group (*P* < 0.001). Compared with the OVA challenge group, IL-17A levels were reduced in the mice of the 20 mg/kg stigmasterol group (*P* < 0.05); IL-17A in the mice of the 10 mg/kg and 20 mg/kg stigmasterol groups, but not statistically significant (*P* > 0.05). The levels of IL-10 in BALF, serum, and splenocytes samples of mice in the OVA challenge group were significantly decreased compared with the control group (*P* < 0.01). Compared with the OVA challenge group, IL-17A levels were reduced in mice in the 20 mg/kg stigmasterol group (*P* < 0.05); IL-17A in mice in the 10 mg/kg and 20 mg/kg stigmasterol groups, but not statistically significant (*P* > 0.05) (Figure 3A–3C).

### Stigmasterol alleviated overexpression of TGF-β1/Smad2 and IL-17A in splenocytes

**Figure 4:**
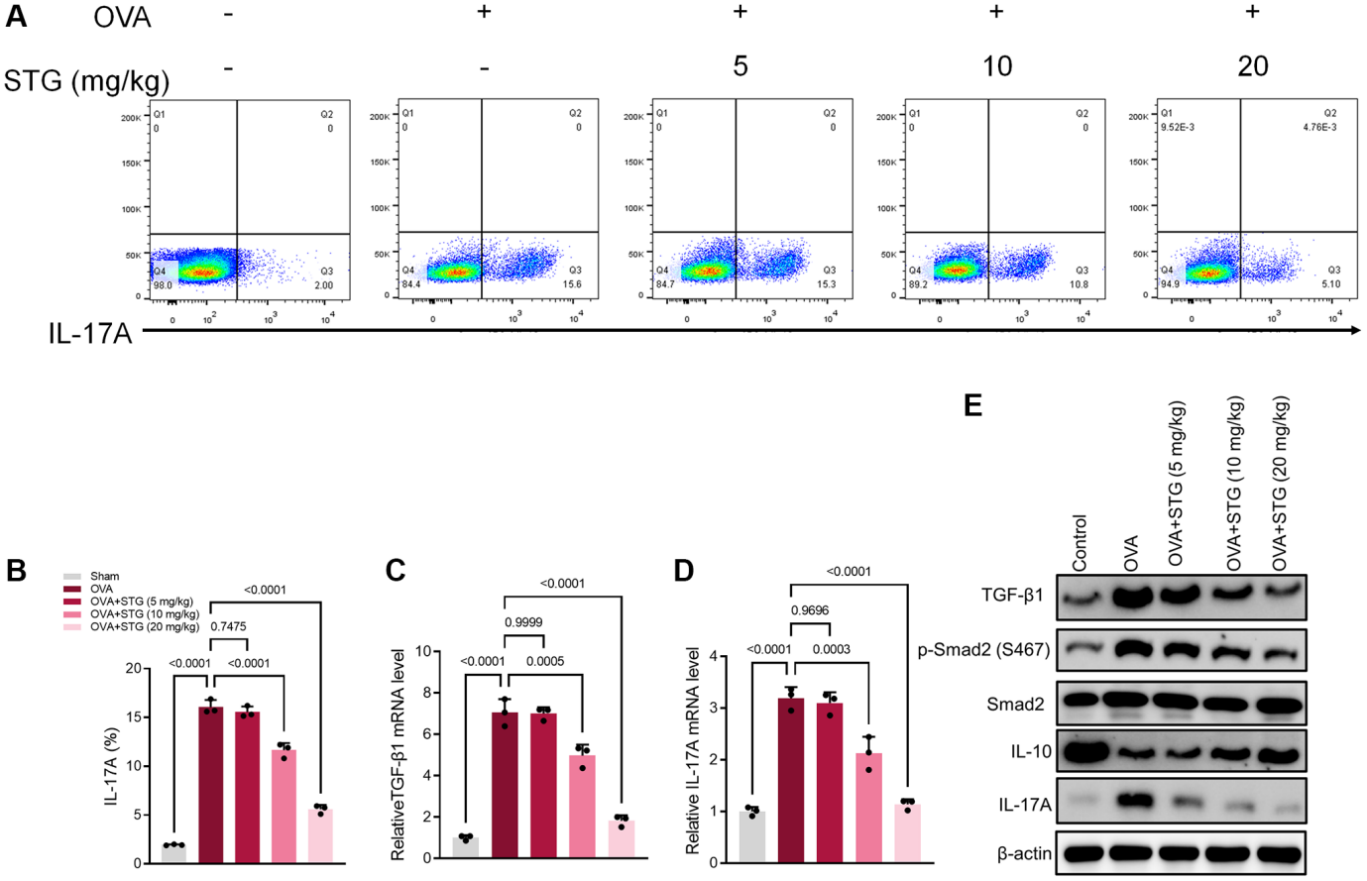
**Differential activation of the TGF-β1/Smad2 and IL-17A signaling pathway in splenocytes.** (**A**) Flow cytometry results showing expression levels of IL-17A in splenocytes. (**B**) The plot of IL-17A levels in splenocytes. (**C**) Differential expression of TGF-β1 gene in splenocytes. (**D**) Differential expression of IL-17A gene in splenocytes. (**E**) Differential expression of TGF-β1, p-Smad2, and IL-17A proteins in splenocytes. *p* < 0.05 indicated statistically significant differences. *p* < 0.05 indicated statistically significant differences.

The percentage of Th17 cells was significantly higher in OVA-challenged mice compared with the Sham group. Within a certain concentration range, the percentage of Th17 cells decreased continuously with the increase in the concentration of stigmasterol, which appeared statistically significant (*p* < 0.001) in the 10 mg/kg and 20 mg/kg stigmasterol groups, compared with the OVA group (Figure 4A, 4B). QRT-PCR of splenocytes revealed that treatment with 10 mg/kg and 20 mg/kg stigmasterol significantly reversed the excessive production of TGF-β1 and Smad2 caused by the OVA challenge (*p* < 0.001), as demonstrated in (Figure 4C, 4D). As expected, compared with OVA group, the protein expression of TGF-β1, p-Smad2, and IL-17A decreased gradually after stigmasterol treatment in a dose-dependent manner. IL-10, an anti-inflammatory cytokine, caused opposite effects on protein expression to those of IL-17A (Figure 4E).

### Stigmasterol reversed TGF-β and IL-6-induced differentiation of CD4^+^ T cells to Th17 cells

**Figure 5:**
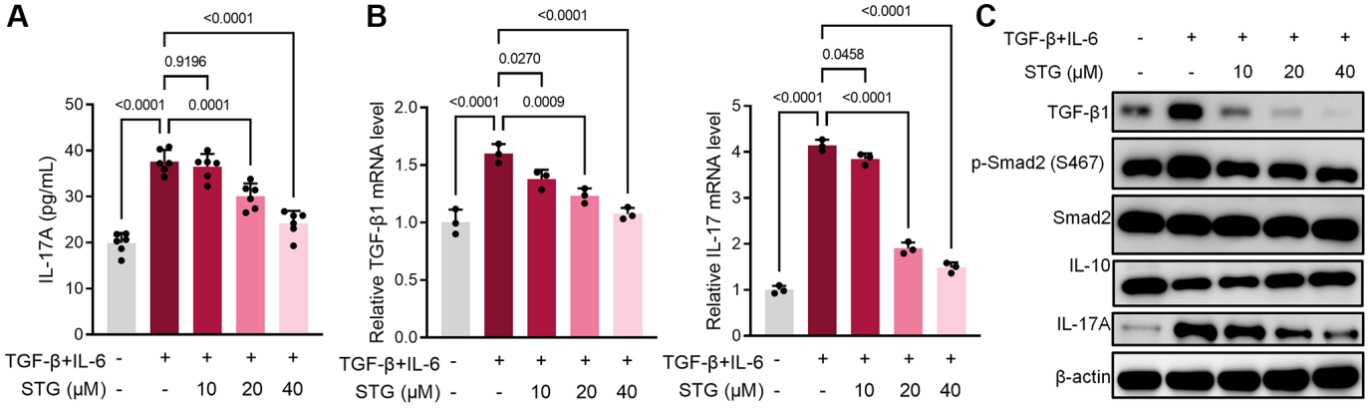
**Effect of stigmasterol on CD4^+^ T cell differentiation.** (**A**) Expression levels of IL-17A in CD4^+^ T cell supernatants. (**B**) Expression level of TGF-β1 and IL-17A gene in CD4^+^ T cells. (**C**) Differential expression of TGF-β1, p-Smad2 and IL-17A proteins in CD4^+^ T cells. *p* < 0.05 indicated statistically significant differences.

CD4^+^ T cells were cultured *in vitro* and differentiated to Th17 cells by TGF-β and IL-6 [22, 23]. After 24 h treatment or absence with stigmasterol. IL-17A level in cell supernatant as significantly upregulated (*p* < 0.001) after TGF-β and IL-6 stimulation, which demonstrated the successful differentiation of CD4^+^ T cells to Th17 cells (Figure 5A). After 10 mg/kg, 20 mg/kg stigmasterol treatment, significant down-regulation was observed. The cells were subjected to QRT-PCR which revealed that 10 mg/kg, 20 mg/kg stigmasterol treatments downregulated expression levels of TGF-β1 and IL-17A (*p* < 0.05), as shown in the Figure 5B. At protein levels, TGF-β1, Smad and IL-17A were down-regulated after stigmasterol treatment. And there was a correlation with the concentration. In contrast, IL-10, an anti-inflammatory cytokine, appeared to be significantly upregulated in the stigmasterol group (Figure 5C).

## DISCUSSION

In recent years, the prevalence of asthma has been on the increase worldwide. Among respiratory problems, asthma is one of the most widespread diseases, which affects about one-third of the world's population. Nearly 2.5 million patients die each year due to severe deterioration of the condition [24]. Currently, the main treatment agent for asthma is glucocorticoids [25]. However, glucocorticoids also present many adverse effects in long-term. It affects musculoskeletal, gastrointestinal, cardiovascular, endocrine systems, skin, ophthalmology, among other organs. Moreover, its effects cannot be easily reverses [26, 27]. Therefore, the search for safe and effective drugs for the prevention and treatment of asthma has become particularly important.

In addition to Th1 and Th2 cells, Th17 cells are also involved in the progression of several immune-mediated lung diseases. Increasing evidence has shown that Th17 cells secrete several types of cytokines (such as IL-17A and IL-6) to exert pro-inflammatory effects in autoimmune diseases, including asthma. Furthermore, abnormally increased IL-17 promote excessive production of eosinophils and neutrophils *in vivo* [28]. Undoubtedly, it has been confirmed that inhibition of IL-17 can suppress eosinophil infiltration [29]. In addition, the report indicates that TGF-β also interferes with the normal remodeling process of asthma airways [30].

In the current experiment, we used OVA-challenged C57 mice, a classical model of asthma. The inhibitory effect of stigmasterol on airway inflammation was demonstrated. As expected, stigmasterol suppressed the excessive production in macrophages, lymphocytes, neutrophils, and eosinophils in BALF. The lymphocyte is mainly found in the lymphatic fluid circulating in the lymphatic vessels. It is important in the immune response of the body. The production of inflammatory factors such as IL-6 and IL-1β by activated macrophages, eosinophils and neutrophils influence the differentiation of Th0 cells to Th17 cells to affect immune function [31]. Significant inflammatory cell infiltration in the lungs was observed in the airways of OVA-induced asthmatic mice. Pharmacodynamics showed that stigmasterol caused a significant decrease in Th17-related cytokines by testing the indicators of BALF. Moreover, it improved the symptoms of AHR. In chronic asthma, allergens and inflammatory cytokines from the lungs would enter the arteries and veins along the capillaries, causing systemic immune system damage. The spleen, as a vital immune organ, would also secrete a large number of T cells to participate in this process [32]. Both IL-10 and IL-17 can be secreted by CD4^+^ T cells, while IL-10 and IL-17 have opposing effects, with IL-10 playing a major anti-inflammatory role [33]. The levels of IL-17A were increased and that of IL-10 were decreased in BALF, serum, and splenocytes in OVA-treated mice, which matched our hypothesis that the increase in Th0 cells is accompanied by excessive differentiation of Th17 cells. The balance between IL-17 and IL-10 was disrupted, causing immune function imbalance. Fortunately, the concentration of stigmasterol at 10 mg/kg and 20 mg/kg had a significant inhibitory effect on IL-17A while upregulating IL-10 expression level. Many studies have shown that TGF-β can promote the differentiation of Th17 cells. TGF-β binds to the corresponding TGF-β receptor on lymphoid T cells. TGF-β acts as a receptor complex formed by ligands that activate phosphorylation of Smad2 into the nucleus and co-activate or repress transcription of target genes. Our study provides key evidence that excessive activation of the TGF-β/Smad pathway may lead to overproduction of IL-17A in OVA-induced asthma [18].

To further test the direct effect of stigmasterol on the differentiation of CD4 T cells in splenocytes, CD4^+^ T cells in splenocytes were isolated to conduct culture experiments. TGF-β and IL-6 were used to induce differentiation of Th17 cells. It was found that stigmasterol significantly inhibited IL-17A levels after 24 h of culture, compared with TGF-β + IL-6 group, which also implies that stigmasterol reduced CD4^+^ T cell differentiation to Th17 cells and reduced inflammation production in splenocytes. Subsequently, proteins from cultured cells were extracted and assayed. Similarly, the results showed that stigmasterol reduced IL-17A levels and promoted IL-10 expression *in vitro* via inhibiting the TGF-β/Smad2 signaling pathway [34].

This study explored the pharmacological mechanism of stigmasterol anti-asthma both *in vivo* and *in vivo*. The experimental data were substantial and meaningful. Although the mouse model of asthma in OVA-challenged mice was relatively mature [35], there was a degree of difference between mouse asthma models and asthma patients. To ensure the feasibility of using IL-17 as a therapeutic target for the future clinical treatment of asthma, more experiments on the safety of stigmasterol need to be designed subsequently. In addition, our findings provide a potential basis for exploring new therapeutic approaches for the treatment of asthma [36].

## CONCLUSION

In summary, the present results demonstrate that stigmasterol have anti-inflammatory effects both locally and systemically, reduces the excessive production of Th 17 cells in the body, increases IL-10 levels and down-regulates IL-17A levels, to improve the symptoms of asthma in mice. These results were also verified through *in vitro* experiments. This study provides a feasible option for the clinical treatment of asthma.
